# 601 Bromelain Based Enzymatic Debridement Followed by Application of Autologous Cell Suspension for Treatment of Burns

**DOI:** 10.1093/jbcr/irac012.229

**Published:** 2022-03-23

**Authors:** Ashley Williams, Maryann Mbaka, Kate Ledbetter, Wilson Huett, Jon D Simmons, Andrew Bright

**Affiliations:** University of South Alabama, Spanish Fort, Alabama; University of South Alabama, Mobile, Alabama; University of South Alabama, 2451 University Hospital Dr, Alabama; University of South Alabama, Mobile, Alabama; University of South Alabama, Mobile, Alabama; USA Health University Hospital, Mobile, Alabama; University of South Alabama, Stockton, Alabama

## Abstract

**Introduction:**

Bromelain based enzymatic debridement has been shown to have a unique selectivity for nonviable tissue and is being evaluated for its use in the treatment of select burn wounds. The treatment has potential to augment more traditional operative eschar excision. Herein, we retrospectively review our tertiary burn center’s early experiences in cases in which bromelain based enzymatic debridement (BBED) is paired with autologous cell suspension (ACS).

**Methods:**

Patients whose burn wounds were deemed eligible for BBED at our institution from July 2020 to June 2021 were queried for concurrent treatment with ACS. Inclusion criteria by study design consisted of 18 years of age or older, non-electrical and non-chemical burns, predominance of deep partial and/or full thickness burn, and less than 30% total body surface area. Success of escharotomy by BBED, grafting methods, infections, transfusions, inflammatory response, and response to grafting were analyzed.

**Results:**

Seven patients met inclusions criteria from July 2020 to June 2021. The mean age was 44 (IQR: 39-53). The mean TBSA was 13.7% (IQR 8-20). The mean time from injury to BBED was 2.1 days (IQR 1.5-2.0). The mean time to ACS after BBED was 1.3 days (IQR 1.0-1.5). The mean area debrided with BBED was 2347 sq cm (IQR 1367-3534). All patients were determined to have complete eschar removal on the day of application by the multidisciplinary burn team. All seven cases had deep partial thickness areas treated with ACS alone, with a mean area of 1574sq cm (IQR 877-2327). The total area treated was 11,016 sq cm. Only two patients required delayed grafting, a combined total of 1573 sq cm, meaning that 86% of the total burn area treated with ACS alone was recovered. Five patients had ACS paired with split thickness autografts, with a mean area of 867 sq cm (IQR 519-1328). Graft imbibition was noted to be rapid with >95% wound closure by postoperative day eight. There were no graft infections, and no transfusions required. White blood cell (WBC) counts decreased after BBED, then increased marginally after grafting. Only two patients had WBC >20,000 and this aided in diagnosis of one donor site infection and one pneumonia.

**Conclusions:**

In our series, BBED within three days of injury paired with ACS within two days of BBED resulted in excellent split thickness graft take and recovery of 86% of the burn wounds treated with ACS alone.

